# HIV-associated mortality in the era of antiretroviral therapy scale-up – Nairobi, Kenya, 2015

**DOI:** 10.1371/journal.pone.0181837

**Published:** 2017-08-02

**Authors:** Peter W. Young, Andrea A. Kim, Joyce Wamicwe, Lilly Nyagah, Catherine Kiama, John Stover, Johansen Oduor, Emily A. Rogena, Edwin Walong, Emily Zielinski-Gutierrez, Andrew Imbwaga, Martin Sirengo, Timothy A. Kellogg, Kevin M. De Cock

**Affiliations:** 1 Division of Global HIV & Tuberculosis (DGHT), U.S. Centers for Disease Control and Prevention (CDC), Nairobi, Kenya; 2 National AIDS and STI Control Programme, Ministry of Health, Nairobi, Kenya; 3 Field Epidemiology Training Program, Ministry of Health, Nairobi, Kenya; 4 Avenir Health, Glastonbury, CT, United States of America; 5 Division of Forensic and Pathology Services, Ministry of Health, Nairobi, Kenya; 6 University of Nairobi and Kenyatta National Hospital, Nairobi, Kenya; 7 Kenya National Bureau of Statistics, Nairobi, Kenya; 8 University of California San Francisco, Nairobi, Kenya; University of Cape Town, SOUTH AFRICA

## Abstract

**Background:**

Declines in HIV prevalence and increases in antiretroviral treatment coverage have been documented in Kenya, but population-level mortality associated with HIV has not been directly measured. In urban areas where a majority of deaths pass through mortuaries, mortuary-based studies have the potential to contribute to our understanding of excess mortality among HIV-infected persons. We used results from a cross-sectional mortuary-based HIV surveillance study to estimate the association between HIV and mortality for Nairobi, the capital city of Kenya.

**Methods and findings:**

HIV seropositivity in cadavers measured at the two largest mortuaries in Nairobi was used to estimate HIV prevalence in adult deaths. Model-based estimates of the HIV-infected and uninfected population for Nairobi were used to calculate a standardized mortality ratio and population-attributable fraction for mortality among the infected versus uninfected population. Monte Carlo simulation was used to assess sensitivity to epidemiological assumptions. When standardized to the age and sex distribution of expected deaths, the estimated HIV positivity among adult deaths aged 15 years and above in Nairobi was 20.9% (95% CI 17.7–24.6%). The standardized mortality ratio of deaths among HIV-infected versus uninfected adults was 4.35 (95% CI 3.67–5.15), while the risk difference was 0.016 (95% CI 0.013–0.019). The HIV population attributable mortality fraction was 0.161 (95% CI 0.131–0.190). Sensitivity analyses demonstrated robustness of results.

**Conclusions:**

Although 73.6% of adult PLHIV receive antiretrovirals in Nairobi, their risk of death is four-fold greater than in the uninfected, while 16.1% of all adult deaths in the city can be attributed to HIV infection. In order to further reduce HIV-associated mortality, high-burden countries may need to reach very high levels of diagnosis, treatment coverage, retention in care, and viral suppression.

## Introduction

Access to antiretroviral treatment (ART) has increased from 1 million to 17 million people living with HIV (PLHIV) globally from 2003 to 2015 [1, 2]. High HIV burden countries have accounted for the majority of this scale-up, with the number of PLHIV on ART increasing from 4 million to over 10 million in the last 5 years in Eastern and Southern Africa [3]. In both high- and low-income countries ART substantially increases life expectancy, especially for patients who start ART early [4, 5]. Given the rapid increase in coverage of life-saving treatment, a commensurate decrease in mortality in HIV-infected populations in high-burden countries would be expected. While cohort studies have demonstrated reductions in mortality among patients accessing ART, high rates of loss to follow-up (LTFU) make it difficult to fully assess the impact of ART at the population level. Complementary surveillance systems that can systematically assess population impact of ART programs on all-cause mortality on an ongoing basis are needed.

Civil registration systems in developing countries are often incomplete and causes of death poorly characterized. While a large fraction of PLHIV may be enrolled in care, high levels of LTFU, undocumented transfers, and passive reporting of deaths mean that program data underestimate mortality in the population in care if not linked to vital statistics [6] or adjusted statistically with LTFU studies [7, 8]. Hospital-based surveillance can shed light on HIV mortality among hospitalized patients, when both testing and HIV surveillance are routinely conducted [9]. Disease case surveillance systems, which could link patient records across systems, are still in their infancy in these settings. Further, the population in care is likely to have reduced mortality compared with those not in care, due to having access to medical interventions.

In urban areas where a majority of cadavers pass through mortuaries, mortuary-based studies have the potential to contribute to our understanding of excess mortality among HIV-infected persons. Although the literature is sparse on use of HIV testing in mortuary settings for surveillance purposes, De Cock *et al*. conducted a mortuary-based study to estimate mortality rates due to HIV and AIDS in Abidjan, Cote D’Ivoire in the late 1980s, before effective treatment for HIV existed, demonstrating that HIV was the leading cause of death in the city at the time [10]. A similar study conducted in 2001 found that nearly half of adult deaths in Pointe-Noire, Republic of Congo could be attributed to HIV, while the City of New York has been routinely testing for HIV during autopsies since the early 1990’s [11]. In Addis Ababa, Ethiopia, mortality surveillance at burial grounds demonstrated reductions in AIDS-related mortality following scale-up of ART [12].

Coverage of ART has rapidly expanded in Kenya, reaching 59% of all PLHIV by the end of 2014 [3], and is likely to be highest in the capital city of Nairobi. The high estimated coverage of death registration in Nairobi at 78% in 2014 [13], together with the fact that all registered deaths in Nairobi are notified by mortuaries, implies that mortuary-based surveillance could be representative of all deaths in the city. Relatively high ART coverage among PLHIV coupled with high levels of mortuary-based death registration and moderate HIV prevalence in adults, made Nairobi an attractive site for examining HIV-related adult mortality. We conducted a cross-sectional mortuary-based serological study to measure HIV prevalence among deaths in Nairobi. We used results from this study to estimate the population-level association between HIV and mortality for the city of Nairobi in the era of scale-up of ART towards universal access.

## Materials and methods

### Mortuary HIV surveillance

The two largest mortuaries in Nairobi in terms of admissions volume were selected for HIV mortuary surveillance. Kenyatta National Hospital (KNH) is a national teaching and referral hospital and its mortuary saw 9,272 admissions in 2014. In contrast, City Mortuary saw 5,253 admissions in 2014 and primarily receives cadavers brought by the police in addition to cases from smaller health facilities lacking a mortuary. All cadavers admitted to KNH and City Mortuary over a 33 day period from 29 January to 3 March 2015 were registered. Cadavers of persons aged 15 years and above at death were sampled using transthoracic aspiration of cardiac blood which was shipped within 6 hours to a central laboratory for processing. Plasma was tested using the national HIV diagnostic algorithm. Specimens were screened with Colloidal Gold assay (KHB Shanghai Kehua Bio-Engineering Co, LTD, Shanghai, China). Reactive results were confirmed using First Response HIV 1-2-0 assay (PMC Medical India Pvt Ltd, Mumbai, India). Specimens with discrepant results were tested with Uni-Gold *HIV* assay as a tie breaker (Trinity Biotech PLC, Bray, Ireland) and the result of the tie-breaker was taken as the final result for the specimen. Every seventh specimen that was non-reactive on the screening assay was confirmed as an internal quality assurance step.

### Nairobi Civil registration

To determine the proportion of annual deaths reported during the study period for City Mortuary, KNH, and city-wide, as well as the proportion of deaths reported by each mortuary and seasonal variation of reported deaths, we abstracted death notifications from the Vital Statistics office at Nairobi City Hall for adults aged 15 and above at time of death for all deaths reported in Nairobi in 2014. We extracted summary data of total deaths by age, sex and year for Nairobi from the 2014 annual vital statistics report for purposes of estimating the age/sex distribution of deaths in Nairobi. These data were used to assess the coverage of death reporting in Nairobi as well as the coverage of City and KNH mortuaries among all reported deaths.

### Measures

Age and sex of cadavers in the mortuary study were based on the reported age and sex from the death notification, the sex, date of birth and age recorded in the patient medical records if the death occurred at Kenyatta National Hospital and medical records were available for abstraction, or the autopsy report completed by the pathologist. Age and sex of deaths reported to vital statistics were based on the age and sex recorded on the death notification.

### Population projections

Spectrum version 5.52b3 (Avenir Health, Glastonbury, Connecticut) was used to estimate ART program coverage, HIV-related mortality, as well as the age- and sex-specific HIV prevalence for Nairobi in 2015, based on official demographic statistics, HIV surveillance and program data and population-based survey data for Nairobi [14–17]. The age and sex distribution of HIV prevalence from Spectrum was applied to the sub-national population projection for Nairobi from the Kenya National Bureau of Statistics [18] to estimate the number of people living with and without HIV in Nairobi in 2015. A life table derived from the Spectrum projection for Nairobi was used to estimate age-specific mortality rates. The projection is summarized in S1 Table.

### Analysis methods

Data were logged into an Epi Info version 7.0.4 database (U.S. Centers for Disease Control and Prevention [CDC], Atlanta). Data cleaning, tabulation and statistical analyses were performed in Stata version 13 (Stata Corp., College Station, Texas). Coverage and representativeness of surveillance system deaths were assessed by plotting reported versus expected deaths by year and by age group and sex. Inverse probability weights were developed and used to adjust the observed deaths from the surveillance system to account for missing HIV status by age and sex, and mortuary population weights were developed to adjust for differences in age- and sex-specific coverage and annualize deaths with respect to expected deaths based on the KNBS population projection and the Spectrum life table. Mortality rates were calculated by dividing the weighted annualized deaths from the surveillance system by the estimated population size in Nairobi from the population projection, by age, sex and HIV status, then multiplying by 1000.

The standardized risk difference subtracts the mortality rate in the HIV-uninfected population from the corresponding rate in the HIV-infected population, by age and sex. It is an additive measure which describes the additional mortality burden in the HIV-infected population. The standardized mortality ratio (SMR) is a relative measure which divides the mortality rate in the infected population by the mortality rate in the uninfected population after standardizing to the age and sex distribution of the infected population. Both measures were calculated using Stata’s epitab package.

The population attributable fraction (PAF) estimates the proportion of all deaths that would have been avoided if HIV were no longer a risk factor for mortality in the HIV-infected. Odds ratios were fit using logit models with population frequency rates normalized to the total observed deaths, and these were then used to calculate attributable fractions with Stata’s punaf command [19]. We also used logit models to test for interaction between age group and sex.

### Simulation methods

Monte Carlo simulations were conducted to assess the sensitivity of risk ratio and attributable fraction estimates to epidemiological assumptions; additional details are provided in S1 Text.

### Data access

Data are available from the National AIDS & STI Control Programme (NASCOP), Ministry of Health, Kenya on request. Requests for access should be directed to head@nascop.or.ke.

### Ethics approvals

This public health surveillance activity was approved by the U.S. Centers for Disease Control Center for Global Health as research that did not involve human subjects (because subjects were deceased) and did not require institutional review board review for human experimentation [2014/062]. The Kenyatta National Hospital/University of Nairobi Ethical Review Committee also approved this study [P380/06/2014] and did not require consent from next of kin. All data on deceased subjects were de-identified prior to data analysis.

## Results

We found a pooled, unadjusted HIV positivity rate of 19.5% (95% CI 16.5–22.9%) among deaths at KNH and City Mortuaries, the positivity differing significantly by sex (14.6% among men, 29.5% among women, p<0.001). After weighting to account for missing test results and the coverage of the included mortuaries with respect to expected deaths by age and sex, the estimated HIV positivity among adult deaths in Nairobi was 20.9% (95% CI 17.7–24.6%) (Table 1).

**Table 1:** Percent HIV positivity among cadavers at City and KNH mortuaries, Nairobi 2015.

Sex	Age (years)	Unadjusted HIV-positivity (n = 610)	(95% CI)	Adjusted HIV-positivity (n = 807)*	(95% CI)
Male	15–24	8.5	(3.2,20.6)	8.9	(3.3,21.7)
	25–44	15.3	(11.1,20.8)	15.3	(11.1,20.8)
	≥45	15.5	(10.5,22.3)	15.8	(10.7,22.7)
	Total	14.6	(11.5,18.4)	14.8	(11.6,18.7)
Female	15–24	40.0	(19.1,65.4)	40.8	(19.6,66.1)
	25–44	46.1	(36.0,56.5)	46.1	(35.8,56.7)
	≥45	12.5	(7.2,20.8)	12.4	(7.2,20.7)
	Total	29.5	(23.5,36.2)	30.2	(24.0,37.3)
	Grand-total	19.5	(16.5,22.9)	20.9	(17.7,24.6)

Notes

* Adjusted for missing HIV test results and coverage of selected mortuaries by age and sex, assuming HIV status missing at random.

A total of 807 deaths of persons aged 15 years and above (range 15–105 years, median 40 years) were reported during the 33 day period of data collection at the two mortuaries. These corresponded to 8,320 deaths when annualized and seasonally-adjusted. Based on a demographic projection, there were 16,173 deaths of persons aged 15 years and above for Nairobi City in 2015, indicating that after annualizing, the coverage of the sampled mortuaries was 65.0% of reported deaths, and 51.4% of expected deaths. Coverage versus expected deaths varied substantially by age and sex from 22.2% for women aged 15–24 years to 63.5% for men aged 25–44 years. Annualized deaths were then adjusted for this age- and sex-specific coverage to obtain the total adjusted deaths (Table 2).

**Table 2:** Annualized and coverage-adjusted surveillance deaths, Nairobi 2015.

Sex	Age (years)	Observed mortuary deaths *	Annualized mortuary deaths †	Registered deaths in Nairobi ‡	Estimated coverage of registered deaths	Expected deaths for Nairobi §	Estimated coverage of expected deaths	Adjusted mortuary deaths ¶
Male	15–24	61	629	846	74.3%	1,059	59.4%	53
	25–44	277	2,856	3,124	91.4%	4,497	63.5%	225
	45+	197	2,031	3,552	57.2%	4,168	48.7%	209
	Total	535	5,515	7,522	73.3%	9,724	56.7%	486
Female	15–24	19	196	491	39.9%	881	22.2%	42
	25–44	123	1,268	2,076	61.1%	2,681	47.3%	134
	45+	130	1,340	2,707	49.5%	2,887	46.4%	144
	Total	272	2,804	5,274	53.2%	6,449	43.5%	321
	Grand-total	807	8,320	12,796	65.0%	16,173	51.4%	807

Notes: Some totals do not match sum of rows due to rounding errors.

* Deaths registered at KNH and City Mortuaries during the study period.

† Deaths from study sites annualized based on seasonally-adjusted study period of 0.097 years.

‡ Deaths registered at Nairobi City Hall in calendar year 2014.

§ Expected deaths based on Kenya National Bureau of Statistics (KNBS) projection and Spectrum life table for Nairobi.

¶ Deaths annualized and adjusted for differential coverage by age and sex with respect to expected deaths for Nairobi.

An estimated 2.8 million adults aged 15 years and above were residents of Nairobi in 2015, corresponding to 160,386 HIV-infected and 2,649,060 HIV-uninfected adults based on an overall HIV prevalence of 5.6% for Nairobi (Table 3). Dividing the estimated deaths from the surveillance system by the total projected population, the crude mortality rate in Nairobi among persons aged 15 years and older was 5.76 / 1,000 persons. In women, age-specific mortality estimates ranged from 1.92 / 1,000 among those aged 15–24 to 16.70 / 1,000 among women aged 45 and above. In men, mortality ranged from 3.16 / 1,000 among men aged 15–24 to 17.41 among men aged 45 and above. Overall, the mortality rate among HIV-infected persons was 21.12 / 1,000 compared with 4.83 / 1,000 among HIV-uninfected persons aged 15 years and older. Among HIV-infected women the mortality rate was 20.59 / 1,000 compared with 21.89 / 1,000 among HIV-infected men (Table 3). The standardized mortality ratio was 4.35 (95% CI 3.67–5.15), indicating that risk of death was significantly higher among HIV-infected persons compared with HIV-uninfected persons, when controlling for age and sex. The standardized risk difference was 0.016 (95% CI 0.013–0.019), corresponding to 16 additional deaths per 1,000 population among the HIV-infected after adjusting for age and sex (Table 4). The interaction between age and sex was significant, so all estimates were calculated using models stratified by sex and age group.

**Table 3:** Estimated age- and sex-specific deaths, population, and mortality rates per 1000 population, Nairobi 2015.

	Age (Years)	HIV-negative	HIV-positive	Total
Sex		deaths / pop (rate)	deaths / pop (rate)	deaths / pop (rate)
Male	15–24	967/330,458 (2.93)	95/6,011 (15.80)	1,062/336,469 (3.16)
	25–44	3,818/767,352 (4.98)	691/46,156 (14.97)	4,509/813,508 (5.54)
	45+	3,518/226,141 (15.56)	660/13,885 (47.53)	4,178/240,026 (17.41)
	Total	8,303/1,323,951 (6.27)	1446/66,052 (21.89)	9,749/1,390,003 (7.01)
Female	15–24	499/424,695 (1.17)	344/13,607 (25.28)	843/438,302 (1.92)
	25–44	1,449/737,582 (1.96)	1238/70,315 (17.61)	2,687/807,897 (3.33)
	45+	2534/162,832 (15.56)	360/10,412 (34.58)	2,894/173,244 (16.70)
	Total	4,482/1,325,109 (3.38)	1,942/94,334 (20.59)	6,424/1,419,443 (4.53)
Overall		12,785/2,649,060 (4.83)	3,388/160,386 (21.12)	16,173/2,809,446 (5.76)

**Table 4:** Risk difference (RD), standardized mortality rate (SMR) and population attributable fraction (PAF) due to HIV, Nairobi 2015.

	Sex					
	Male		Female		Total	
Statistic	Estimate	(95% CI)	Estimate	(95% CI)	Estimate	(95% CI)
SMR	3.12	(2.43–3.99)	6.17	(4.86–7.83)	4.35	(3.67–5.15)
RD	0.015	(0.010–0.020)	0.017	(0.013–0.021)	0.016	(0.013–0.019)
PAF	0.101	(0.067–0.133)	0.254	(0.199–0.305)	0.161	(0.131–0.190)

Combining the risk estimates with the prevalence of HIV in the population, the population attributable fraction (PAF) was 0.161 (95% CI 0.131–0.190). The estimated PAF differed between men and women, with women having a higher PAF for HIV infection than men: the PAF for women was 0.254 and for men was 0.101 (Table 4).

There were 40,382 males and 77,640 females aged 15 years and above on ART in Nairobi by December 31, 2015 according to official HIV program statistics. The Spectrum ART coverage estimate for 2015 for all HIV-infected persons aged 15 or more years in Nairobi was 73.6%, 61.1% for males and 82.3% for females, and Spectrum predicted that 13.8% of male deaths and 8.3% of female deaths (11.4% combined) would have been HIV-infected in 2015 (S1 Table).

Our sensitivity analysis looked at several key parameters that were estimated for this analysis as presented in S2 Table. The outcomes were most sensitive to HIV prevalence in the mortuaries and in the population, as well as the ratio of observed to unobserved prevalence in the mortuaries. The uncertainty interval for the SMR obtained through the uncertainty analysis was 3.04–7.19, while for the PAF the uncertainty interval was 0.118–0.221 (S3 Table).

## Discussion

We conducted an analysis to combine HIV prevalence in a sample of deaths in Nairobi with population estimates in order to assess the burden of mortality associated with HIV in adults. We believe this is the first African study of its kind in the era of ART scale-up and as such it provides important insights in to the association of mortality and HIV infection in a major urban center with generalized HIV prevalence and a public ART program that reaches the majority of adults living with HIV. People living with HIV are at risk from death from multiple competing causes, including HIV. By estimating measures such as the SMR, RD and PAF this study looks beyond disease prevalence and provides estimates of the continuing impact of HIV on the adult population of Nairobi. Despite 7 in 10 HIV-infected persons aged 15 years and above receiving ART, our study showed persons living with HIV had a four-fold increased risk of mortality compared with those not infected. The population attributable fraction of 0.161 shows that about one in six deaths would be avoided in Nairobi if the increased mortality associated with HIV infection were eliminated, and more deaths would potentially be avoided in women than in men.

Antiretroviral treatment has been shown to dramatically reduce premature mortality. A prior study in Central Africa conducted at the height of the HIV epidemic when HIV prevalence was similar to Nairobi, but before ART was widely available, found nearly half of adult deaths were HIV-infected, while an East African cohort study in the late 1990’s found a 20-fold increased mortality risk among the HIV-infected before widespread access to ART. A national study in South Africa found consistent declines in population all-cause mortality associated with early expansion of ART access from 2005–2009. In our study, conducted after over a decade of ART scale-up, we found about 1 in 5 deaths was HIV-infected, yet people living with HIV continue to be at significantly increased risk of death with a greater than four-fold increased risk of mortality. This is especially true for women in Nairobi who have reached 82.3% ART coverage in 2015 –surpassing the UNAIDS fast-track target of 81% of all PLHIV on treatment.

This study had several limitations. The mortality data were collected in early 2015, but the distribution of deaths available for abstraction at the time of the study was for 2014. The coverage of the two participating mortuaries was moderate, but may not have been representative of all deaths in the city. The calculation of mortality rates assumes that measured deaths were from the resident population, however deaths in Nairobi are not necessarily drawn from the resident population of the city. Some Nairobi residents die outside the city limits, and some non-Nairobi residents die within the city. The latter group is likely to be over-represented at KNH since it is a national referral hospital and routinely receives patients transferred from other counties.

Although these two facilities do not include all deaths in the city, they do include a significant fraction of deaths that occur in Nairobi. Although there may be some seasonal factors that affect mortality, Nairobi does not generally suffer from seasonal malaria epidemics or other infectious disease outbreaks that are likely to be over- or under-represented during the study period. After seasonal and annual adjustments for total deaths, our study covered 51.4% of expected deaths based on demographic estimates, but covered 65.0% of reported deaths, but coverage varied substantially by age and sex. Our sensitivity analysis found that outcome measures were sensitive to assumptions about HIV prevalence and representativeness of sampled mortuaries, however our uncertainty analysis showed that overall conclusions were robust in the face of this uncertainty, lending confidence in the robustness of the findings that the risk of dying is greater in the HIV-infected population than in the HIV-uninfected population, and that a considerable fraction of deaths at the population level can be attributed to HIV in Nairobi.

Though a lower proportion of HIV-infected men access treatment, their lower HIV prevalence and competing causes of mortality result in HIV being a less frequent cause of death. A complete diagnosis, treatment and adherence history for all deaths could help elucidate to what extent undiagnosed infection, inadequate treatment access or late presentation, poor adherence, or treatment failure are responsible for the mortality differentials seen among PLHIV. Mathematical modeling has shown that the majority of deaths among PLHIV are likely to be among those not on treatment, even in high ART coverage settings, underscoring the need to reduce barriers to treatment initiation and improve retention on ART. Thus it was surprising to find a higher HIV-associated SMR among women than men, in spite of their greater access to ART. This may be due to over-representation of deaths due to external causes in our sample, which differentially affect men. If this is the case, our estimates of HIV mortality among men are conservative. Inclusion of additional mortuaries in future studies would help control this potential bias. Despite study limitations, it appears that mortality among PLHIV in Nairobi continues to be high in spite of high estimated ART treatment coverage. This high mortality among PLHIV may help explain why HIV prevalence has not increased in recent years in spite of continued new infections and a rapid expansion of public HIV care and treatment services [20].

## Conclusion

Although sub-Saharan Africa has dramatically decreased mortality due to HIV through expansion of ART, in order to further reduce HIV-associated mortality, high-burden countries may need to reach very high levels of diagnosis, treatment coverage, retention in care, and viral suppression. Though hospitals are no longer overwhelmed with AIDS patients, Kenya has not yet achieved the goal of an AIDS-free generation.

## Supporting information

S1 Text Simulation methods. (DOCX)

S1 Fig Scatter plot matrix showing pairwise correlation between statistics (PAF and SMR) and input parameters, Nairobi 2015. (DOCX)

S1 Table Summary of demographic and epidemic projection, population age 15 years and above, Nairobi 2015. (DOCX)

S2 Table Parameters used for Monte Carlo simulation-based sensitivity and uncertainty analyses for standardized mortality ratio (SMR) and population attributable fraction (PAF), Nairobi 2015. (DOCX)

S3 Table Simulation results for overall and sex-specific standardized mortality ratio (SMR) and population attributable fraction (PAF), Nairobi 2015. (DOCX)
