# Supplementary material for: HIV-associated mortality in the era of antiretroviral therapy scale-up – Nairobi, Kenya, 2015
Source: PLoS One. 2017 Aug 2;12(8):e0181837. doi: 10.1371/journal.pone.0181837 (PMC5540587; doi:10.1371/journal.pone.0181837)
Supplement: S1 Text — (DOCX) [file pone.0181837.s001.docx]

# S1 Text. Simulation Methods

Monte Carlo simulations were conducted to assess the sensitivity of risk ratio and attributable fraction estimates to epidemiological assumptions (HIV prevalence in the mortuaries, in the population, coverage of the sampled mortuaries with respect to all actual deaths, and the impact of seasonality of overall death notifications at sampled mortuaries, the male/female population prevalence ratio, and the ratio of observed to unobserved HIV prevalence in mortuaries in Nairobi). Input parameters are summarized in S2 Table. Each input parameter was independently sampled 10,000 times in Stata from a normal (log-normal in the case of ratio measures) random distribution. The mean was set to the “best” value and the standard deviation chosen to cover alternative plausible values for the parameter. Through trial and error 10,000 runs were selected to ensure the Monte Carlo standard error, a measure of the accuracy of the simulation itself, was less than 2.5% as reported by Stata’s simsum command. R version 3.1.2 (R Development Core Team) was used to generate scatter plot matrices to visualize associations between input and output parameters. Non-parametric uncertainty bounds were generated for outcomes using the 2.5^th^ and 97.5^th^ centiles from the resulting distribution. Unlike traditional confidence intervals which describe uncertainty due to random sampling, the uncertainty bounds also reflect uncertainty about model assumptions. Results are summarized in S3 Table.
