# Supplementary material for: HIV-associated mortality in the era of antiretroviral therapy scale-up – Nairobi, Kenya, 2015
Source: PLoS One. 2017 Aug 2;12(8):e0181837. doi: 10.1371/journal.pone.0181837 (PMC5540587; doi:10.1371/journal.pone.0181837)
Supplement: S1 Fig — (DOCX) [file pone.0181837.s002.docx]

# Figure S1. Scatter plot matrix showing pairwise correlation between statistics (PAF and SMR) and input parameters, Nairobi 2015


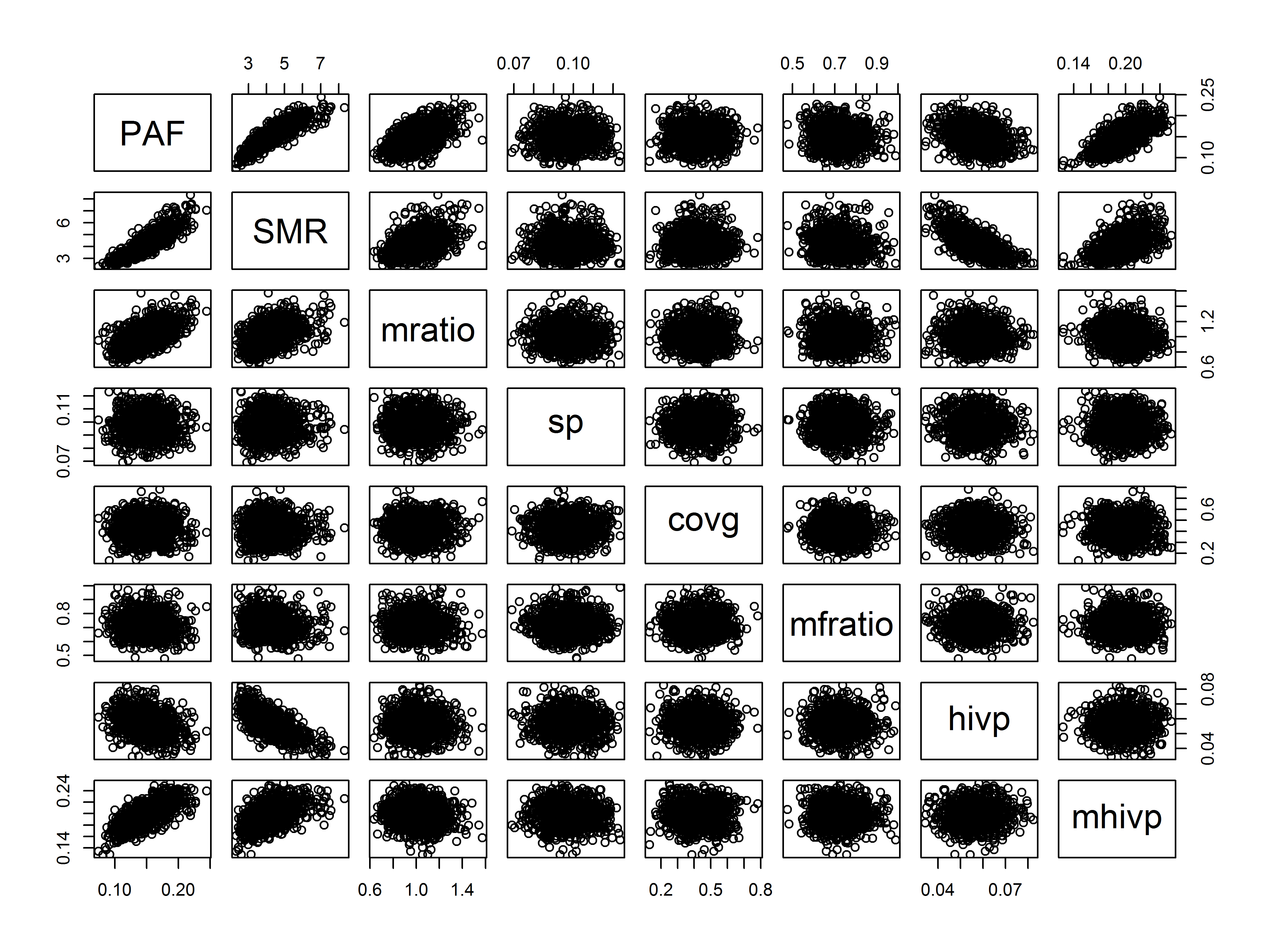
Definitions: PAF = population-attributable fraction, SMR = standardized mortality ratio, mratio = ratio of prevalence among all deaths vs sampled mortuaries, sp = study period, coverage = coverage of included mortuaries, mfratio = male/female HIV prevalence ratio in the population, hivp = population HIV prevalence, mhivp = mortuary HIV prevalence. The figure shows an inverse relationship between SMR and population HIV prevalence, and positive association between PAF and mortuary HIV prevalence and PAF and the ratio of prevalence among all deaths to sampled deaths. Other modeled inputs have less or no impact on estimated SMR and PAF.
