# Supplementary material for: HIV-associated mortality in the era of antiretroviral therapy scale-up – Nairobi, Kenya, 2015
Source: PLoS One. 2017 Aug 2;12(8):e0181837. doi: 10.1371/journal.pone.0181837 (PMC5540587; doi:10.1371/journal.pone.0181837)
Supplement: S1 Table — (DOCX) [file pone.0181837.s003.docx]

# Table S1. Summary of demographic and epidemic projection, population age 15 years and above, Nairobi 2015

| Indicator | Total | Men | Women |
| --- | --- | --- | --- |
| Population* | 2,809,448 | 1,390,003 | 1,419,445 |
| PLHIV† | 160,386 | 66,051 | 94,335 |
| Number on ART (coverage)‡ | 118,022 (73.6%) | 40,382 (61.1%) | 77,640 (82.3%) |
| All deaths§ | 16,173 | 9,724 | 6,449 |
| % deaths HIV-infected | 11.4% | 13.8% | 8.3% |
| Standardized mortality ratio (SMR) due to HIV║ | 3.84 | 6.64 | 1.91 |
| Population-attributable fraction (PAF) due to HIV¶ | 0.081 | 0.117 | 0.035 |

Notes: * Total 2015 mid-year population and population age distribution from Kenya National Bureau of Statistics (KNBS) projections. † age-specific and total 2015 mid-year PLHIV from combining KNBS projected population with Spectrum age- and sex-specific HIV prevalence distribution. ‡ Number on ART from official country program statistics as reflected in Spectrum (numbers on ART and ART coverage reported for December 31). § Deaths obtained by applying mortality rates from Spectrum life table for Nairobi to KNBS projected population for 2015.║Standardized mortality ratio calculated by indirectly standardizing mortality rate in the HIV-negative population as estimated by Spectrum by 5-year age groups to the sex and age distribution among the HIV-infected, as estimated by Spectrum. ¶ Population-attributable fraction calculated by multiplying the proportion of deaths HIV-infected according to Spectrum by (SMR-1)/SMR.
