# Supplementary material for: HIV-associated mortality in the era of antiretroviral therapy scale-up – Nairobi, Kenya, 2015
Source: PLoS One. 2017 Aug 2;12(8):e0181837. doi: 10.1371/journal.pone.0181837 (PMC5540587; doi:10.1371/journal.pone.0181837)
Supplement: S2 Table — (DOCX) [file pone.0181837.s004.docx]

# Table S2. Parameters used for Monte Carlo simulation-based sensitivity and uncertainty analyses for standardized mortality ratio (SMR) and population attributable fraction (PAF), Nairobi 2015

| Parameter | Mean | Sd |
| --- | --- | --- |
| HIV prevalence | 0.563 | 0.008445 |
| Mortuary HIV prevalence | 0.2133659 | 0.018062 |
| Mortuary prevalence ratio | 1.0 | 0.15 |
| Male/female ratio | 0.71501239 | 0.107 |
| Sampling period | 0.097 | 0.0093 |
| Mortuary coverage | 0.512 | 0.1 |
