# Supplementary material for: HIV-associated mortality in the era of antiretroviral therapy scale-up – Nairobi, Kenya, 2015
Source: PLoS One. 2017 Aug 2;12(8):e0181837. doi: 10.1371/journal.pone.0181837 (PMC5540587; doi:10.1371/journal.pone.0181837)
Supplement: S3 Table — (DOCX) [file pone.0181837.s005.docx]

# S3 Table. Simulation results for overall and sex-specific standardized mortality ratio (SMR) and population attributable fraction (PAF), Nairobi 2015

| Statistic | Median | 95% UI | MCSE |
| --- | --- | --- | --- |
| SMR | 4.58 | (3.04-7.19) | 1.051% |
| SMR (male) | 3.52 | (2.31-5.60) | 0.830% |
| SMR (female) | 6.53 | (4.31-10.37) | 1.534% |
| PAF | 0.167 | (0.118-0.221) | 0.019% |
| PAF (male) | 0.120 | (0.075-0.160) | 0.016% |
| PAF (female) | 0.268 | (0.205-0.337) | 0.024% |

Notes: The Monte Carlo sampling error (MCSE) is a measure of the precision of the simulation and is a function of number of repetitions and variability in the simulated statistic. The number of repetitions was selected to ensure an MCSE less than 2.5% for each statistic for each sub-group (male, female or combined). The 95% uncertainty interval (2.5^th^ to 97.5^th^ centiles) summarizes the variability in the estimate that is due to the uncertainty in the input parameters as provided in S1 Table. The SMR fell between 3.04–7.19, and the PAF fell within 0.0118–0.221 in 95% of simulations, lending support to the robustness of the finding of increased mortality among PLHIV aged 15 years and older in Nairobi.
